# Crowdsourcing Vector Surveillance: Using Community Knowledge and Experiences to Predict Densities and Distribution of Outdoor-Biting Mosquitoes in Rural Tanzania

**DOI:** 10.1371/journal.pone.0156388

**Published:** 2016-06-02

**Authors:** Stephen Peter Mwangungulu, Robert David Sumaye, Alex Julius Limwagu, Doreen Josen Siria, Emmanuel Wilson Kaindoa, Fredros Oketch Okumu

**Affiliations:** 1 Environmental Health and Ecological Sciences Thematic Group, Ifakara Health Institute, Ifakara, Tanzania; 2 School of Geospatial Science and Technology, Ardhi University, Dar es Salaam, Tanzania; 3 Institute of Tropical Medicine, Antwerp, Belgium; 4 School of Public Health, Faculty of Health Sciences, University of the Witwatersrand, Parktown, South Africa; Institut Pasteur, FRANCE

## Abstract

Lack of reliable techniques for large-scale monitoring of disease-transmitting mosquitoes is a major public health challenge, especially where advanced geo-information systems are not regularly applicable. We tested an innovative crowd-sourcing approach, which relies simply on knowledge and experiences of residents to rapidly predict areas where disease-transmitting mosquitoes are most abundant. Guided by community-based resource persons, we mapped boundaries and major physical features in three rural Tanzanian villages. We then selected 60 community members, taught them basic map-reading skills, and offered them gridded maps of their own villages (grid size: 200m×200m) so they could identify locations where they believed mosquitoes were most abundant, by ranking the grids from one (highest density) to five (lowest density). The ranks were interpolated in ArcGIS-10 (ESRI-USA) using inverse distance weighting (IDW) method, and re-classified to depict areas people believed had high, medium and low mosquito densities. Finally, we used odor-baited mosquito traps to compare and verify actual outdoor mosquito densities in the same areas. We repeated this process for 12 months, each time with a different group of 60 residents. All entomological surveys depicted similar geographical stratification of mosquito densities in areas classified by community members as having high, medium and low vector abundance. These similarities were observed when all mosquito species were combined, and also when only malaria vectors were considered. Of the 12,412 mosquitoes caught, 60.9% (7,555) were from areas considered by community members as having high mosquito densities, 28% (3,470) from medium density areas, and 11.2% (1,387) from low density areas. This study provides evidence that we can rely on community knowledge and experiences to identify areas where mosquitoes are most abundant or least abundant, even without entomological surveys. This crowd-sourcing method could be further refined and validated to improve community-based planning of mosquito control operations at low-cost.

## Background

Mapping distributions of disease-transmitting mosquitoes and monitoring their biting activity can help community members and public health authorities to plan and implement appropriate interventions [1–3]. Geographical Information System (GIS) has become a major tool for the analysis of geo-referenced health related data and its application is likely to increase further. A key area of GIS application is for analysis of disease spread and determinants, mostly to improve management strategies and resource allocation in high-risk areas [4].

Participatory GIS has also been increasingly embraced, thus enabling incorporation of indigenous knowledge into GIS frameworks [5]. This approach allows greater participation and empowers community members and stakeholders who would normally not be considered GIS experts. Previous examples of participatory GIS applications in research and development include planning and resource management in low income countries [6,7], provision of health services [8], physical planning of infrastructure [5,9], monitoring environmental pollution [10] and conflict resolution [11]. It has also been used for policy analysis, interpretation and formulation, as well as for effective community engagement particularly on issues of cultural rights and land ownership in various communities [12–14]. Though the potential of participatory GIS has also been embraced in disease vector monitoring, in most cases, the purpose has been primarily to achieve ground-truthing for remotely collected datasets. Moreover, internet based platforms now allow communities of users to pull their knowledge, experiences and resources in ways far beyond any single individual, a technique often referred to us Crowdsourcing [15,16]. While such online crowdsourcing approaches increase the speed and capabilities with which we can create solutions, they also provide large sets of data, which could be mined additionally to create essential new knowledge and address problems in a highly scalable way [17–19]. Unfortunately, such capabilities are only starting to be exploited against major global health problems [18].

Diseases transmitted by mosquitoes including malaria, yellow fever, dengue and arboviruses still constitute major challenges in sub-Sahara Africa. In Tanzania, malaria alone affects millions and is among leading courses of hospitalization and child deaths. Lymphatic filariasis remains endemic in the country [20] and dengue fever viruses resulted in 1017 cases and 4 deaths in 2014 [21]. Other mosquito-borne infections such as Rift Valley fever [22–24] and Chikungunya have also been documented in some areas [25–27]. Ongoing mosquito control interventions such as long-lasting insecticidal nets (LLINs) and indoor spraying with residual insecticides (IRS) are having significant impact already [28], but the remaining residual transmission which occurs in spatially segregated areas must also be targeted to ensure elimination is achieved [29,30]. To achieve effective control of mosquito-borne infections, surveillance techniques for both the pathogen and the vectors should be improved, so they are readily scalable even in hard-to-reach areas.

The aim of this study was therefore to employ community-based participatory mapping approach to predict densities and distribution of disease-transmitting mosquitoes in remote and rural areas. It was also to demonstrate the value of enhanced participatory GIS, local knowledge and experience in risk mapping exercises. In an initial exploratory study, we tested this idea in a single study village by simultaneously trapping mosquitoes outdoors in 20 different locations in dry season [1]. We also asked community members to rank these areas based on where they thought mosquitoes were most abundant and observed significant similarities between clusters of mosquito biting hotspots as obtained from interpolated community data and concurrent entomological data [1]. In this current study, we also intended to validate these earlier observations, but this time working in 3 different villages (not including the one studied initially). Our main aim was therefore to test this innovative crowd-sourcing approach, which relies simply on knowledge and experiences of residents, as a way to rapidly predict areas where disease-transmitting mosquitoes are most abundant. We have tested this crowd-sourcing approach as a potentially low-cost and scalable approach to improve vector surveillance and control.

We deliberately focused on the risk of outdoor-biting mosquitoes since this is an increasingly important challenge to malaria vector control. Existing mosquito surveillance techniques mostly rely on indoor mosquito collections, and would miss outdoor vectors, which could potentially contribute significantly to the ongoing residual transmission diseases such as malaria [31,32]. It is therefore important to identify new ways to improve surveillance and monitoring of outdoor-biting. The resulting surveillance-response strategy will help optimize geographical allocation of new interventions, so that they target most of the outdoor-biting mosquitoes, thus enhancing effectiveness. It should however be noted that this approach could also be used to target indoor-biting vectors or vectors that are preferentially very domestic, such as *Aedes aegypti*, which transmits dengue fever viruses and several other arboviruses. Using the same techniques, community members could be relied upon to identify known environmental determinants of vector densities, such as improperly discarded used vehicle tires, solid waste damps, and other potential vector breeding habitats

## Methods

### Study area

**Fig 1:**
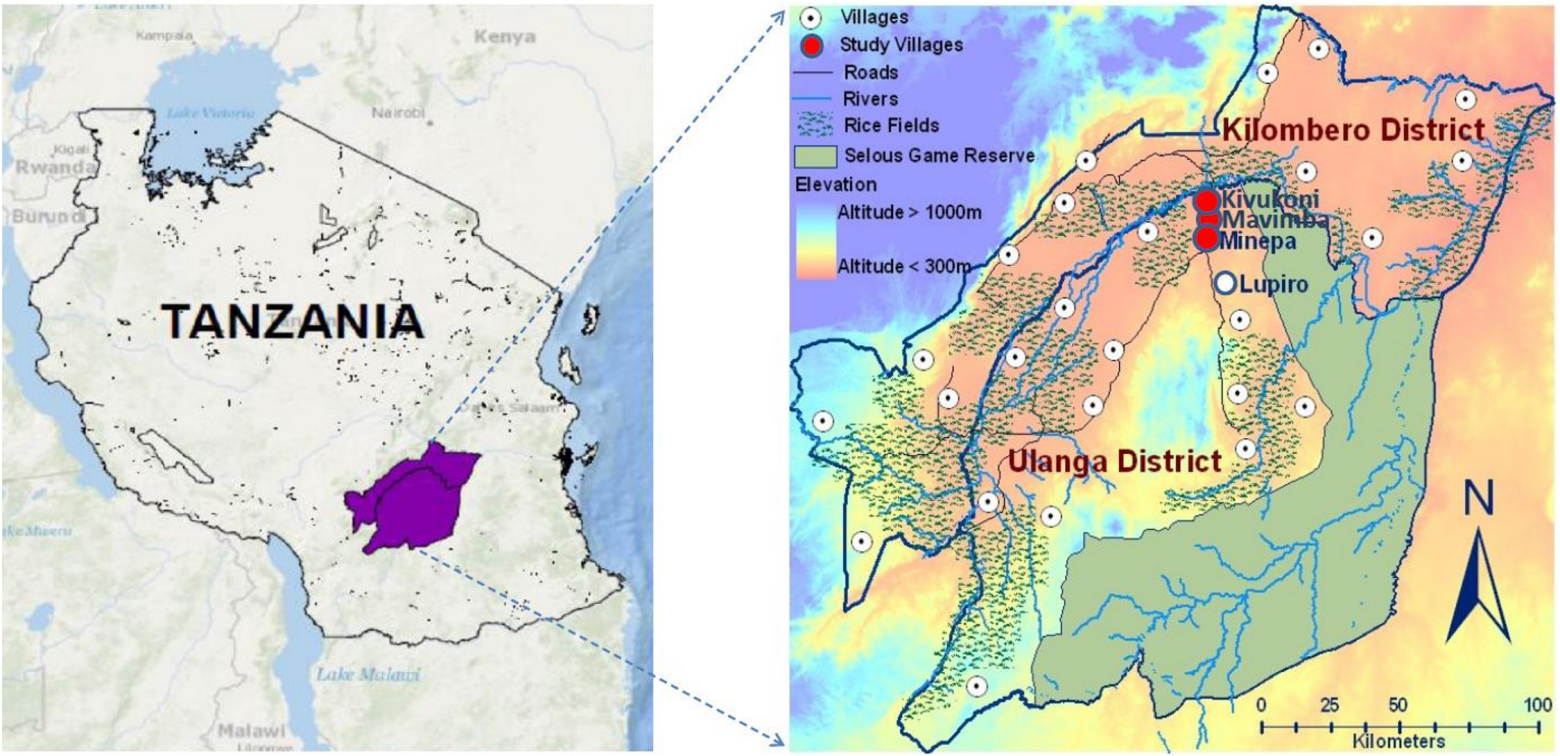
Study areas. Map showing the three villages where the study was conducted (Kivukoni, Minepa and Mavimba) in rural Ulanga district, southeastern Tanzania.

The study was conducted in three villages (Fig 1) namely Kivukoni (8.2135°S, 36.6879°E), Minepa (8.2710°S, 36.6771°E) and Mavimba (8.3124°S, 36.6771°E). All three villages are within the Ifakara Health and Demographic Surveillance System (HDSS) area [33] in Ulanga district, southeastern Tanzania. The villages lie between 120 to 350 meters above sea level on the flood plains of the Kilombero river valley, between the Udzungwa mountain ranges to the northwest and Mahenge hills to the southeast. Annual rainfall ranges between 1200 mm and 1800mm, with short rains peaking between December and January and long rains peaking between March and April. Mean daily temperatures range between 20°C and 32°C, while relative humidity is between 70% and 90% on average. The main economic activity of the inhabitants is subsistence farming, consisting of rice cultivation. A few families also keep livestock to supplement income or practice small-scale fishing in the Kilombero river and its tributaries. Mosquito densities have been historically high in the area, and currently the major malaria vectors include *Anopheles arabiensis* and *Anopheles funestus*. There is widespread use of mosquito nets, which have been either evaluated or widely distributed over several years [34,35]. The population of each of these villages as per the data maintained by the community leaders in 2015 were as follows: Kivukoni (a total of 5,893 people living in 745 households and consisting 2,777 males and 3,116 females), Minepa (a total of 6, 207 people living in 903 households and consisting of 2,796 males and 3,411 females) and Mavimba (a total of 4167 people living in 785 households, and consisting of 1,987 males and 2,180 females).

### Initial mapping of physical features and households in study area

**Fig 2:**
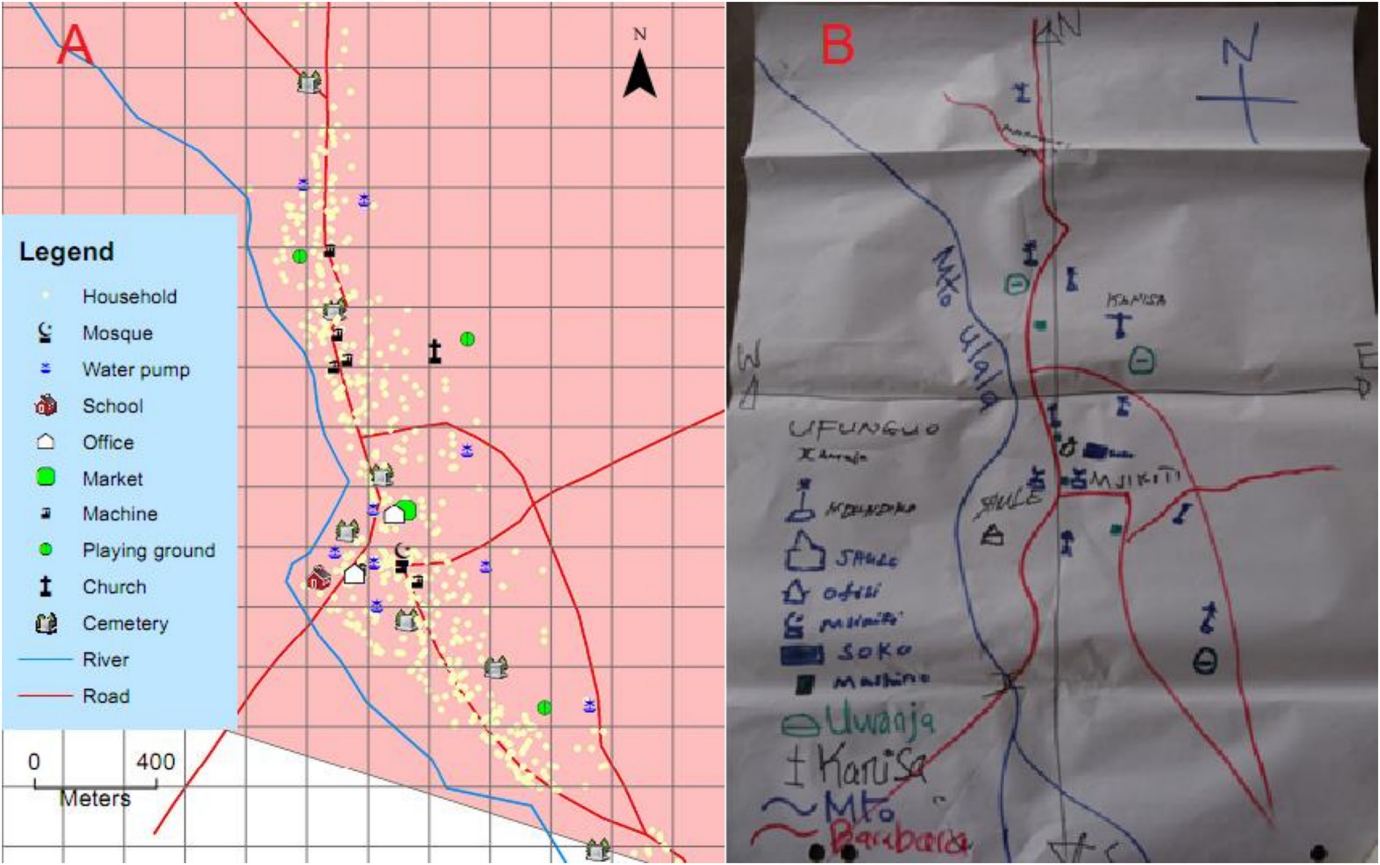
Participatory mapping of study villages. Panel A is an example of expertly produced map of one of the study villages (Kivukoni village), showing the 200m × 200m grids and locations of households and other important features. Panel B shows an example of maps that the community members produced during the participatory mapping sessions.

We initially surveyed the study areas with assistance from volunteer community resource persons, using techniques previously used by Chaki *et al* [36]. We mapped important point and polygon features including religious buildings, grain mills, water pumps and wells, local government offices, rice fields, playing grounds, cemeteries, markets, health centers and schools. Locations of these physical features were captured using handheld global positioning system (GPS) receivers (Magellan eXplorist GC, USA). Important line features, including streams, rivers, main roads and feeder roads were also marked using GPS. The household geo-location data for the three study villages were obtained from Ifakara HDSS [33]. The GPS data was imported into ArcGIS Desktop 10 (ESRI, USA), and used to prepare maps clearly depicting landmark features to aid with map reading and orientation. Each village map was further customized by overlaying small square grids (200m × 200m), after which paper maps of each village were printed for subsequent sessions of map reading (Fig 2A).

### Selection of participants

Beginning July 2012 to June 2013, a total of 720 participants from each of the three study villages involved in the exercise. Each month, we worked with 60 participants, including 20 primary school pupils (11–15 years old), 20 secondary school pupils (16–19 years old) and 20 adults (18 years and older). The participants were selected on a monthly basis per village with intention of having different participants each month throughout the year of the study, all groups consisting of 50% males and 50% females. Selection of participants from the respective study villages was done with help of village leaders, school teachers and community-based volunteers. We recruited only those who had been permanent residents in the respective villages for at least 5 years prior to the study, and were able to read and write in the local language of Kiswahili, and provided written informed consent either from themselves, if adults, or from their guardians, parents or teachers, in the case of non-adults.

### Group discussions and map reading sessions

Once the community participants were selected and written informed consent obtained, we held sessions of guided discussions each month for each of the groups, i.e. adults, primary school-going pupils and secondary school-going children in each of the three villages. In these sessions, research team members first explained the purpose of the study exhaustively to the participants. These interactive sessions were led by a facilitator using a set of pre-tested questions, and consisted of two parts. In the first part, the participants were guided through interactive discussions and hands-on exercises about mosquitoes, diseases they transmit and methods used to control them, while in the second part the participants were guided through further discussions and hands-on exercises on map reading and interpretation, in this case examples from their own villages. Each of the sessions lasted between 60 and 90 minutes. Lay language was used throughout all sessions, using directly observable examples and pictures, and encouraging the participants to practice drawing and interpreting maps features of their villages. The session facilitators ensured no new knowledge on mosquito ecology was introduced, and instead the participants were encouraged to share with one another the knowledge they already had.

In the second session, i.e. the map-reading session, the participants learned and practiced map-reading by sketching maps of their own villages on 1m × 0.6m charts attached to the wall. They were also asked to indicate on these maps some important features, such as roads, religious buildings, rivers, schools and markets (Fig 2B). The exercises were conducted in groups and participants were free to ask one another and exchange ideas. The facilitators ensured that it was highly participatory and that at the end of the sessions, the participants had adequately familiarized themselves with the outlines and key features of their surroundings, and that they were able to represent this in the form of sketch maps (Fig 2B). Using printed maps, the participants also practiced the actual process of map reading, and were guided through basic processes such as directional orientation (North-South-East-West) and how to identify features and approximate distances on maps. They were guided on how to read and interpret features represented on their village maps, using the sketches prepared by the participants themselves, and also expertly produced geo-referenced maps of the villages.

### Stratification of village maps by individual participants to represent their perceived spatial variations of mosquito densities

After the 60–90 minute discussion and map-reading sessions, during which the participants worked in groups, they were then separated and each person individually provided with gridded maps of their respective villages (Fig 2). The participants were asked to identify areas on the maps, where they believed that outdoor-biting mosquito densities were highest and areas where they believed the densities lowest, based on their own experiences and knowledge. They were asked to rank the grids on a scale of 1 to 5, where 1 represented areas with highest outdoor-biting densities and 5 represented areas with lowest outdoor-biting densities of mosquitoes. Participants worked with maps of their own villages, and each person only worked with a single map, on which he or she assigned ranks to different grids, indicating his or her expected mosquito densities in those grids were relative to the other grids on the map. The 3 village maps had 192 grids (Kivukoni village), 192 grids (Minepa) and 221 grids (Mavimba), all grids being 200m × 200m.

Throughout this process, the community members were not required to distinguish between different mosquito taxa or identify mosquitoes of medical importance. We instead focused on perceived outdoor-biting densities by all mosquitoes in general, primarily because community level opinions about vector control are known to depend also on levels of nuisance biting by non-disease vectors [37,38]. Moreover, distinguishing species or taxa was considered unnecessarily technical and beyond the scope of this study.

### Summarizing and interpolating the community knowledge and experiences

Once the community members had ranked the grids on the maps of their villages, we needed to create summarized surface maps to depict areas believed by the community members to have highest densities to those believed to have lowest outdoor-biting densities. The ranking information of specific grids over each village map was therefore summarized in a Microsoft Excel spreadsheet, and assigned quantitative weights to represent values assigned by community members. In a reverse order, the grids ranked 1 were assigned an arbitrary weight of 5, grids ranked 2 were assigned weight of 4 and so on and so forth, so that grids ranked 5 were assigned weight of 1. This reverse weighting was necessary because when we worked with the community members, it was more intuitive for most people to classify places with highest densities as best performing, yet for subsequent statistical interpretation, we needed to represent these densities in a direct linear fashion. For purposes of this analysis, no values were assigned to grids that had not been ranked by the participants. Instead, these grids were considered to have missing values, but not as zero values.

Ranking by all the 60 participants were summarized as follows: The final value, representing community-perceived mosquito abundance, *y*, for each individual grid (*y*_*i*_), was calculated as the sum of the products of weighted ranks assigned to each grid (*w*_*1–5*_) and number of respondents assigning that particular rank to that particular grid (*z*_*1–60*_) as follows:

$$y_{i}=\left(W_{1}\times Z_{w1,i}\right)+\left(W_{2}\times Z_{w2,i}\right)+\left(W_{3}\times Z_{w3,i}\right)+\left(W_{4}\times Z_{w4,i}\right)+\left(W_{5}\times Z_{w5,i}\right)$$ (1)

where *y*_*i*_ refers to the overall community-perceived mosquito abundance in the *i*^*th*^ grid; *z*_*w1*,*i*_, *z*_*w2*,*i*_, *z*_*w3*_, *z*_*w4*,*i*_, and *z*_*w5*,*i*_, refer to the number of times a rank has been assigned to the *i*^*th*^ grid and can take any values form 0 to 60, thus the maximum possible value of *y* is 300, assuming 60 respondents assign a value of 5 to any grid. Values *w*_*1*_, *w*_*2*_,…*w*_*5*_ refer to the weights assigned to each grid on the scale of 1 (lowest) to 5 (highest).

We used the centroids (latitude and longitude of the center of each grid) as the reference points to enable creation of village-wide surfaces containing values representing outdoor mosquito biting densities for all the grids with corresponding coordinates.

This geo-referenced data was then imported into ArcGIS Desktop 10 (ESRI-USA), and geo-processed in the ArcToolbox application. We used Spatial Analyst tools for interpolation with Inverse Distance Weighted (IDW) method, to generate complete surfaces for all the grids over each of the study villages. The conceptual relationship between data points was assumed to be inversely related such that areas nearby each other would be more likely similar than areas far apart, and the distance between neighboring features was determined using Euclidean distance [39]. The outcome surfaces of these interpolations were reclassified into three categories, depicting community-perceived mosquito densities as high, medium and low density categories.

At the end of this process, all the selected sites were visited and characterised based on predominant physical attributes directly observed.

### Entomological assessments to verify vector densities in locations identified by community members as having high, medium or low mosquito densities

We designed a monthly mosquito sampling strategy for the same period, July 2012 to June 2013, aimed at verifying mosquito density categories as determined by community members, every month in each of the three study villages. Once the interpolated maps were reclassified to show areas considered by community members as having high, medium and low outdoor-biting densities each month, we randomly selected two grids with high density, two grids with low density and another two grids with medium density for the entomological surveys. We then located an odour-baited outdoor mosquito trap in each of these sites, so that in each village, we had six traps used every month, to compare actual densities of host-seeking mosquitoes in the areas. Hand-held GPS receivers were used to locate the traps in the selected grids in each study village. Written informed consent was obtained from the land owners or heads of households prior to locating any of the traps.

**Fig 3:**
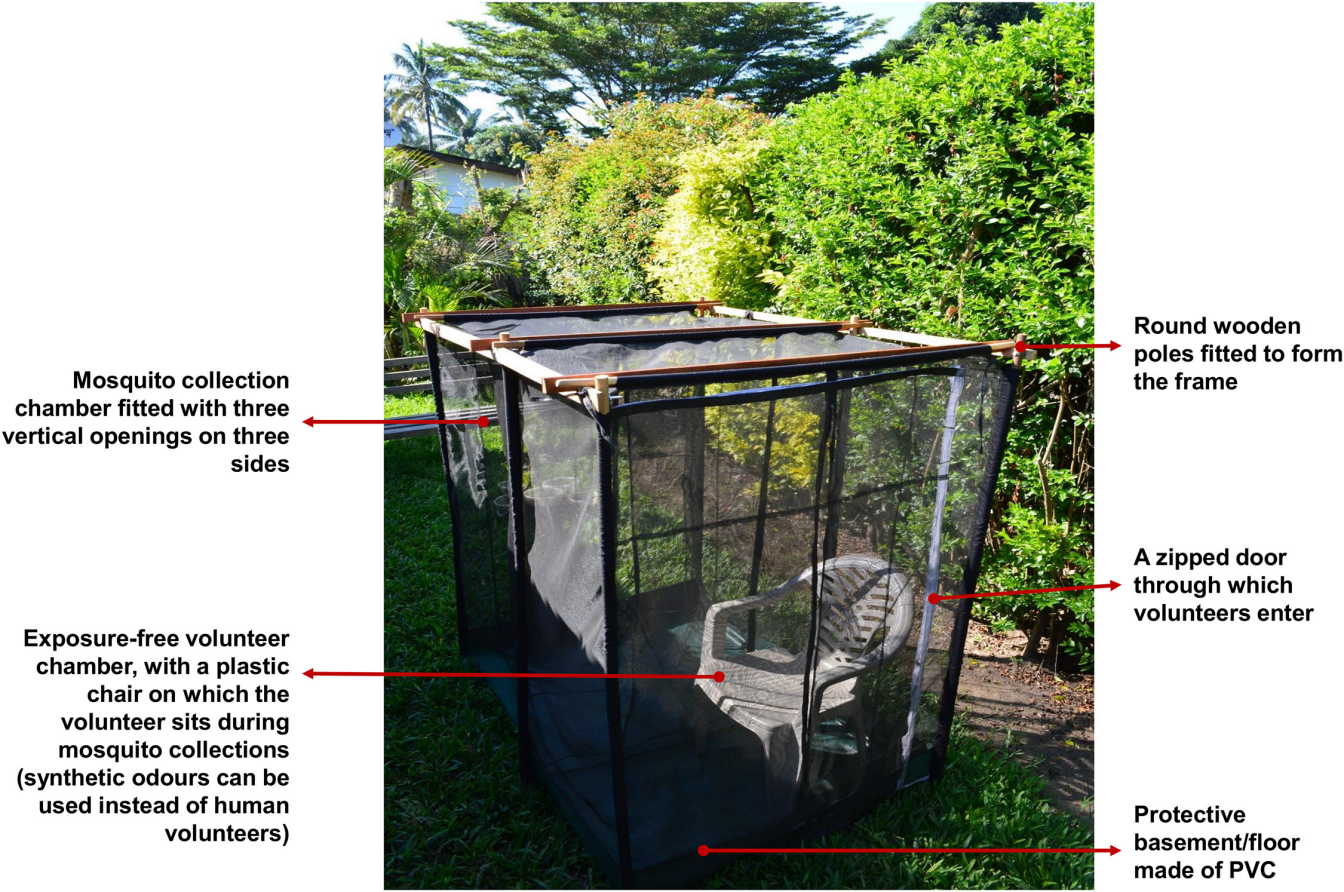
The M-Trap. Pictures of the odour-baited trap, the M-trap, used for comparative assessment of mosquito densities. Vertical envelope-shaped mosquito entry points are labelled. In our study, no human volunteer occupied the trap, and instead we relied on synthetic mosquito attractants complemented with carbon-dioxide gas.

For this purpose, we used a recently developed odour-baited mosquito trap referred to as the M-Trap (Limwagu et al., unpublished). This trap consists of a double-compartment netting cage measuring 120cm × 120cm × 140cm and made of UV-resistant shade netting. It has vertical envelope-shaped mosquito entry points on three of its four sides (Fig 3). The inner compartment is fully enclosed so that mosquitoes entering the trap through the outer envelope-shaped openings cannot reach the inner compartment, inside which the mosquito attractant is located. Complete descriptions of the functionality and efficacy of this trap are provided elsewhere (Limwagu et al., unpublished).

The design of this trap allows working with human volunteers sitting inside the inner segment as baits to attract host-seeking mosquitoes, but without exposing these volunteers to mosquito bites. In this study however, we used a synthetic mosquito lure previously developed by Okumu et al, and dispensed using nylon strips [40,41]. To supplement the synthetic odourants, carbon dioxide (CO_2_) gas, obtained from yeast-sugar fermentation was added, ensuring to use two different yeast concentrations to provide sustained CO_2_ production overnight. [42,43]. In each case, 2 concentrations of yeast-sugar mixture were prepared, one by mixing 30gms of yeast plus 0.125 kg of sugar in 1 litre of clean water, and the other by mixing 20gms of yeast plus 0.125kg of sugar in 1 litre of clean water. The mixtures were prepared 1 hour before start of the experiments. The mosquito attractants including CO_2_ gas were dispensed inside the inner chamber of the M-trap, via 20cm PVC piping fitted with a 12 volt battery-driven fan on the top end to blow the attractants continuously throughout the night [44–47]. Fresh attractants were used in each trap each night. Each morning, an adult trained volunteer entered in the M-traps and collected mosquitoes from the outer chamber, using a mouth aspirator, and kept these mosquitoes separately in properly labelled cups (showing date, village name, trap code, community-perceived density category and individual trap identification code). The traps were located at the centroids of the selected grids, except where there was a physical barrier, e.g. a house, in which case we located the trap right next to the physical barrier. All the traps were located outdoors.

In each study village, two traps were placed in areas of each mosquito density category, as determined based on community knowledge and experiences. These comparative mosquito surveys were done for consecutive 5 nights in each village each month, resulting in fifteen trapping nights per month, from July 2012 to June 2013. To maintain high quality of the data, we separated the tasks such that the entomological surveys were always conducted by a different set of staff and volunteers, independent of the other community members who participated in the group discussions and grid characterization based on knowledge and experiences. For these comparative evaluations, the six traps (2 in each density category) were run simultaneously in each village. We had a total of 18 traps for the three different villages.

### Mosquito identification

Mosquito catches from each trap were identified morphologically and sorted by different taxa (*An*. *gambiae s*.*l*, *An*. *funestus* group, Other *Anopheles species*, *Culex* species and *Mansonia* species). For each taxon, we also classified the mosquitoes as either male or females, and reported the data only for females.

### Analysis of mosquito traps data

Analysis was conducted using an open source statistical package, R [48]. We calculated the median numbers of mosquitoes caught across villages and for all areas identified based on community knowledge and experiences as having high, median and low densities. General linear models (GLM) were fitted and mosquito catches modeled as a function of community perceived mosquito-density categories and months of surveys. We calculated and used median nightly mosquito catches to depict summaries for areas within villages where community members believed there were high, medium or low mosquito densities. Similar summaries were calculated to depict difference between study villages on catches of different mosquito species.

### Ethics statement

Participation in both entomological assessments and the community participatory mapping was voluntary. A detailed explanation of the study aims, as well as potential risks and benefits involved, was provided to all volunteers by the research team prior to any activities. Thereafter, written informed consent was obtained from each individual adult participant. For school children under the age of 18 years, who participated in the study, written informed consent was obtained from the guardians and school teachers. The informed consent forms were prepared in advance and approved together with the complete study protocol, by the institutional and national ethics review boards. To reduce any exposure to mosquito bites, the volunteers involved with mosquito collection were provided with special long sleeved clothing with ventilated hoods to prevent bites during mosquito collection. We used synthetic mosquito attractants and did not rely on any human baited traps in this study, so volunteers in the entomological assessments only went out in the evenings to set up the traps, and then visited the traps in the morning to retrieve the trapped mosquitoes. All volunteers in the entomological assessments also had free access to malaria diagnosis by light microscopy or rapid diagnostic tests, and treatment using the first line drug, artemether lumefantrin (Coartem^™^), if they were found positive for malaria parasite. Fortunately, no volunteer actually was infected with malaria parasite during these experiments. Written informed consent was also obtained from all household heads and land owners in whose compounds we placed mosquito traps during this study. Permission was also obtained from respective village leaders prior to starting the study.

Ethical review and approval was provided by institutional review board of Ifakara Health Institute (Ref: IHI/IRB/NO.030) and Medical Research Coordinating Committee at the National Institute of Medical Research, Tanzania (Ref: NIMR/HQ/R.8a/Vol. IX/1222).

## Results

### Results of crowd-sourcing exercise showing variations of mosquito densities as determined on the basis of community knowledge and experiences

**Fig 4:**
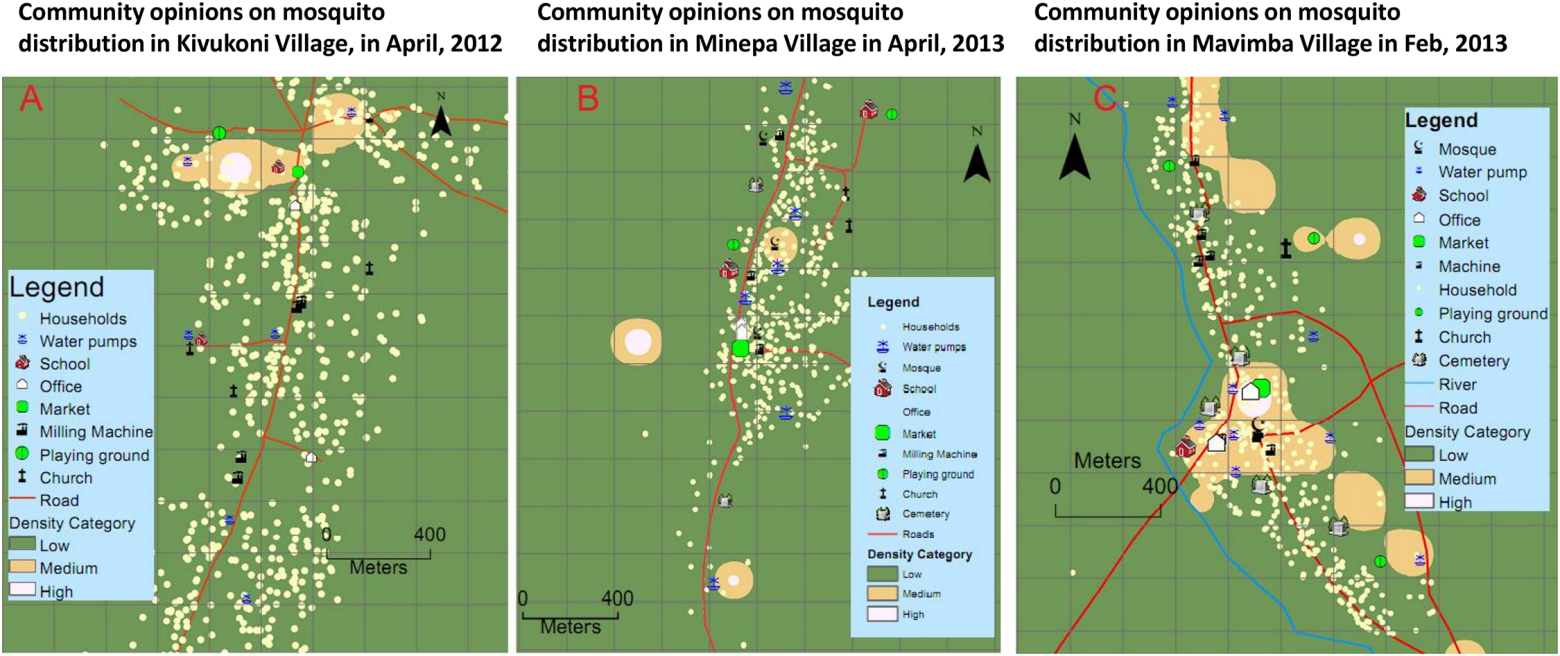
Maps of community opinions. Examples of gridded village maps showing interpolated surfaces of community opinions on where mosquito densities are high, medium or low in Kivukoni, Minepa and Mavimba villages at different times during the study period.

**Fig 5:**
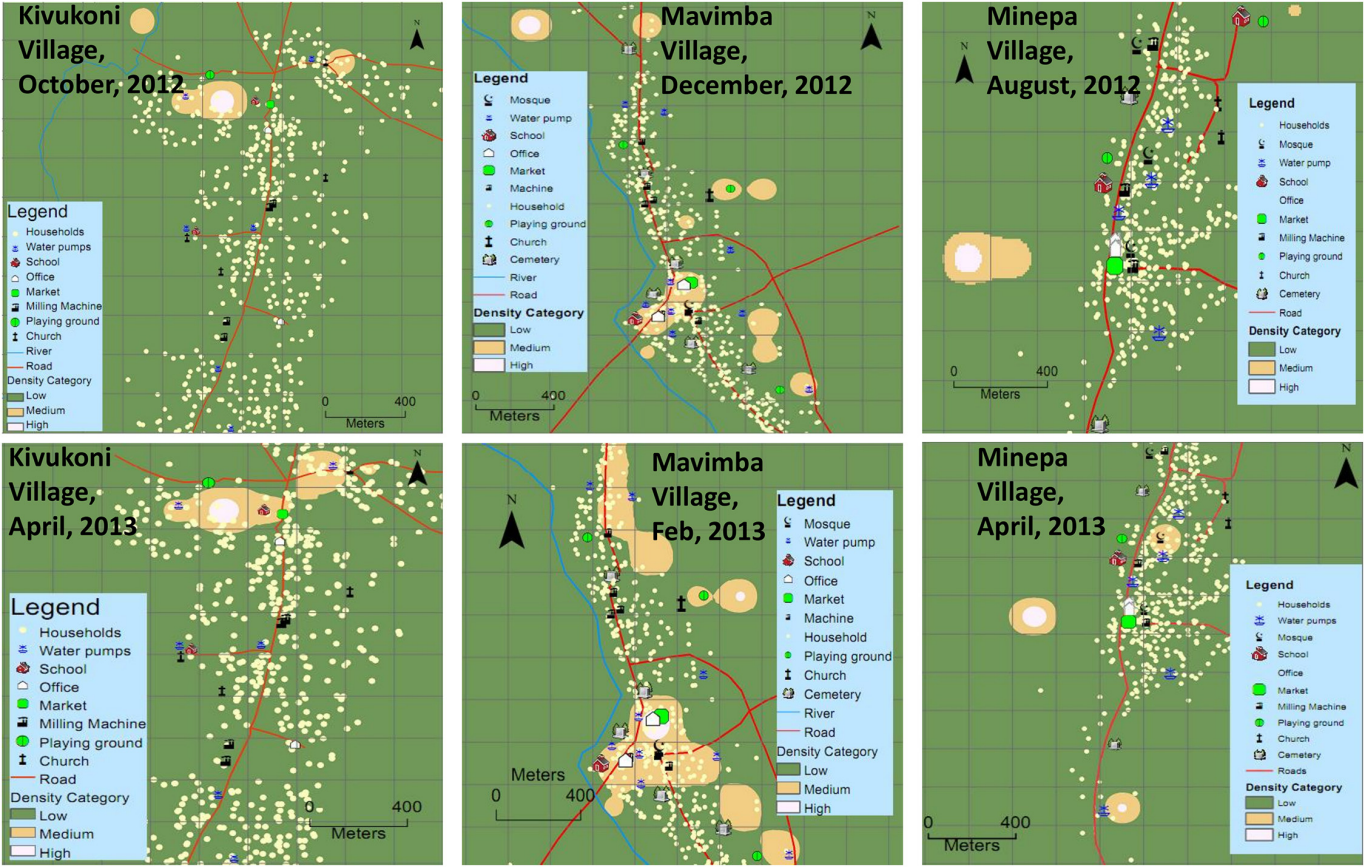
Maps of community opinions in dry and wet seasons. Examples of gridded village maps showing wet season and dry season differences observed on the interpolated surfaces of community opinions on where mosquito densities are high, medium or low in Kivukoni, Minepa and Mavimba villages at different times during the study period.

Examples of interpolated maps of community knowledge and experiences regarding distribution of outdoor-biting mosquito densities are shown in (Figs 4 and 5). Additional maps for all villages during all the months of study are included in the supplementary information (S1 File). The map surfaces shown in Fig 5 for each of the three study villages, are selected to represent data obtained during the period from February to April 2013, which was from the beginning to the peak of the wet season; and for the period from August to December 2012, which was from the peak of dry season to the end of the dry season. Even though the general locations of highest densities of mosquitoes remained the approximately the same throughout the year, there were variations in numbers of community members identifying specific grids as being of highest or medium densities. Fig 5 depicts that the community opinions about areas of highest and medium mosquito densities in all the three villages were more pronounced in wet season than in dry season.

In the first study village (Kivukoni), during the different discussion rounds, opinions of community members suggested that most mosquitoes were found towards the northern part of the village, while southern part of the village had the smallest densities of outdoor-biting mosquitoes (Fig 5A). In the second study village (Minepa), opinions of most participants predicted that mosquitoes were most abundant in southern and western parts of the village, and least abundant in the eastern parts (Fig 5B). For the third study village (Mavimba), community members suggested that most mosquitoes were found in central area, where there was an open market, a mosque, local government offices and highest density of households, while the least densities were believed to occur in western parts of the village (Fig 5C).

### Results of direct observations of physical characteristics of areas determined by community members as having high, medium or low mosquito densities

In all the villages, throughout the 12 months of study, less than 10% of the grids were considered by community members as having high or medium densities of mosquitoes, during the study period. The general physical features we observed when we visited the grids marked by community members as having high, medium and low densities of outdoor-biting mosquitoes are included in Table 1.

**Table 1:** Directly observed physical features predominant in locations marked by community members as having high, medium and low outdoor mosquito biting densites in the three study villages.

Study Village	Community knowledge & experiences	Main physical features observed during field visits
Kivukoni Village	High densities of outdoor biting mosquitoes	a) have high density of households, clustered near the main road and a small fishing camp near the river; b) swampy most of the year, with tall grass and shrubs; c) physical features included a community water pump; d) there is a primary school in the area; d) sampling locations are near road junctions
	Medium Densities of Outdoor Biting Mosquitoes	a) have high density of households, clustered near the main road; b) sampling locations are near road junctions; c) physical features included a community water pump; d) there were 2 markets, a primary school & church in the area; e) all the areas considered as having medium densities were located at the edge of the areas considered to be having high mosquito densities
	Low Densities of Outdoor Biting Mosquitoes	a) has grass and bare land; b) low density of households except near the main road; c) physical features include, rice milling machine, playing ground, community leaders office, water pumps and church
Minepa Village	High densities of outdoor biting mosquitoes	a) the area is extensively cultivated with rice fields divided in small paddocks, and irrigated by hand dug channels running between the paddocks, and b) has low density of households except in one small hotspot in the centre of the village
	Medium Densities of Outdoor Biting Mosquitoes	a) has high density of households in one area but low density of households in the rest; b) other physical characteristic include water pump & mosque; area is isolated and at the age of the areas considered to be having high mosquito densities
	Low Densities of Outdoor Biting Mosquitoes	a) the ground is covered mostly by short shrubs, grass and bare land; b) most grids had no households or low density of households, except near the roads
Mavimba Village	High densities of outdoor biting mosquitoes	a) a very small area in the centre of the areas considered as having medium density of mosquitoes; b) the area has high density of households; c) there is a road junction in the main area considered as having highest mosquito densities; d) other physical facilities were, an open air market, local government offices and mosque in the area
	Medium Densities of Outdoor Biting Mosquitoes	a) has high density of households; b) there is a road junction in the main area considered as having highest mosquito densities; c) other features included, church, milling machines, cemetery, mosque and water pumps; d) there were also open air markets & local government offices in the area
	Low Densities of Outdoor Biting Mosquitoes	a) ground covered mostly by short shrubs, grass and bare land; most grids had no households or low density of households, except near the roads

### Results of entomological assessments of mosquito densities in areas considered by community members as having high, medium or low outdoor-biting densities

**Fig 6:**
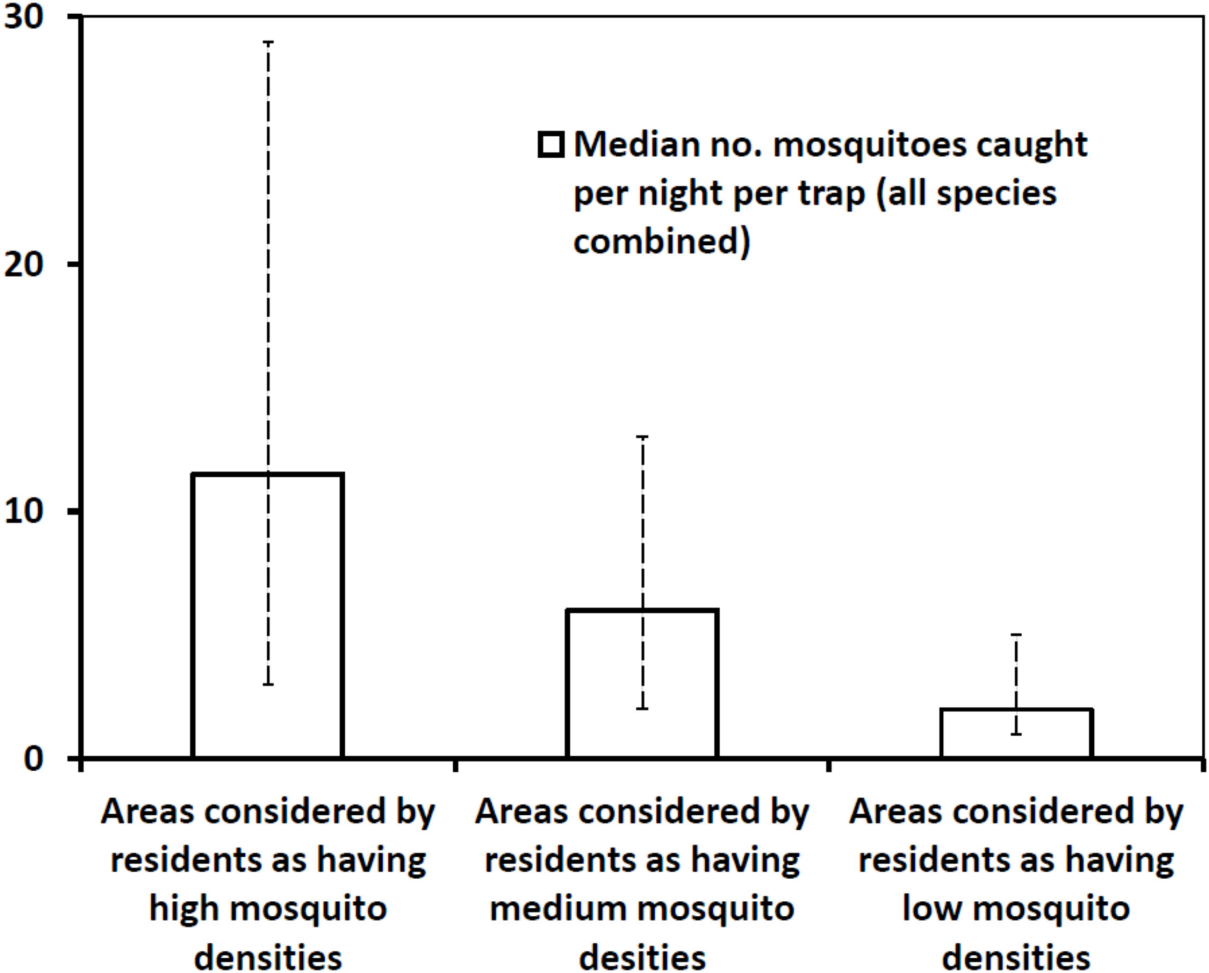
Comparison of mosquito catches in areas classified by communities as having high, medium or low mosquito densities. Overall median mosquito catches in all study villages, in areas marked by community members as having high, medium or low outdoor-biting mosquito densities. Data combined for all mosquito species over 12 months, for all the villages. The error bars in this graph represent the inter-quartile range, i.e. 25^th^ percentile and 75^th^ percentile on either side of the median nightly catch.

A series of comparative monthly mosquito trapping excercises was conducted in areas identifyied by community members every month as having high, medium or low densities of mosquites. The collections were done on a monthly basis for a period of 12 months starting July 2012 to June 2013. Six odour-baited traps were used to trap mosquitoes for 5 consecutive night in each village every month. Overall, we observed that number of mosquitoes of all species combined was highest in areas predetermined by community members as having high densities and lowest in areas predetermined by community members as having low mosquito densities. Significatly more disease-transmitting mosquitoes were caught in areas marked by community members as high density category compared to medium density category and low density category (p < 0.001). Of the 12,412 mosquitoes caught in all the three villages over the duration of the study, 60.9% (7,555) were from areas considered by community members as having high mosquito densities, 28% (3,470) from medium density areas, and 11.2% (1,387) from low density areas. When all mosquito species were combined, the overall median catch (and inter-quartile range) per trap per night was 11.5 (3.3–29.6) in areas marked by community members as having high mosquito densities, 6.1 (2.2–13.5) in areas marked by community members as having median densities, and 2.4 (0.4–5.3) in areas marked by community members as having low mosquito densities (Fig 6).

**Fig 7:**
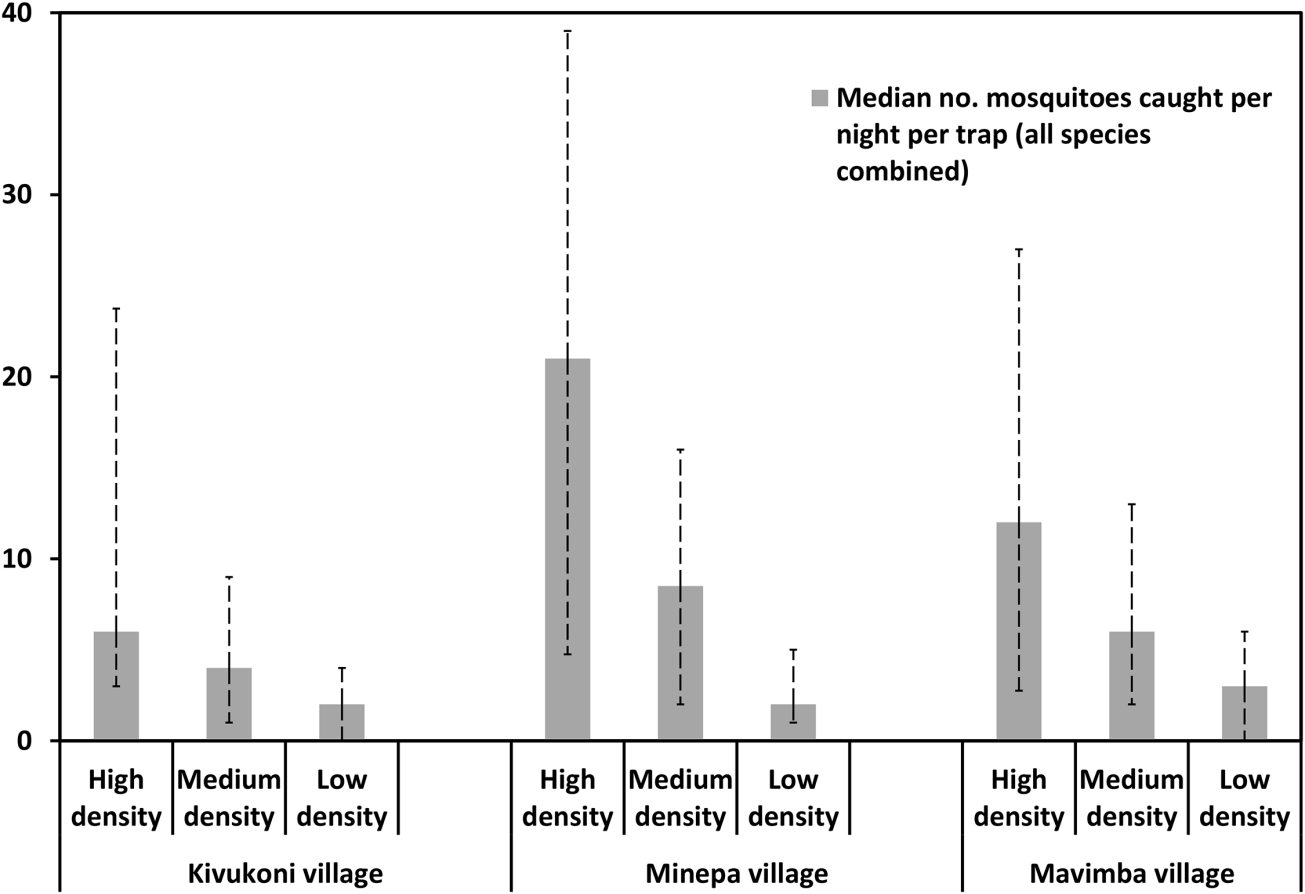
Comparison of mosquito catches in areas classified by communities as having high, medium or low mosquito densities in different villages. Median nightly mosquito catches in areas marked by community members as having high, medium or low outdoor-biting mosquito densities in Kivukoni, Minepa and Mavimba villages. Data combined for all mosquito species over 12 months. The error bars in this graph represent the inter-quartile range, i.e. 25^th^ percentile and 75^th^ percentile on either side of the median nightly catch.

This observation was also clear when data from the individual study villages was considered separately (Fig 7). In all the three different villages, when data was combined for all the 12 months of study, the median mosquito catches per night per trap were always highest in the locations where residents had suggested that the mosquito densities would be highest. Similarly, the median nightly trap catches were lowest in places where opinion of residents had indicated that mosquito densities would be lowest. The results showed that in Minepa village mosquito catches were slightly higher compared to the other two study villages although the trends were same as predicted by opinions of community members from the entire study village.

**Fig 8:**
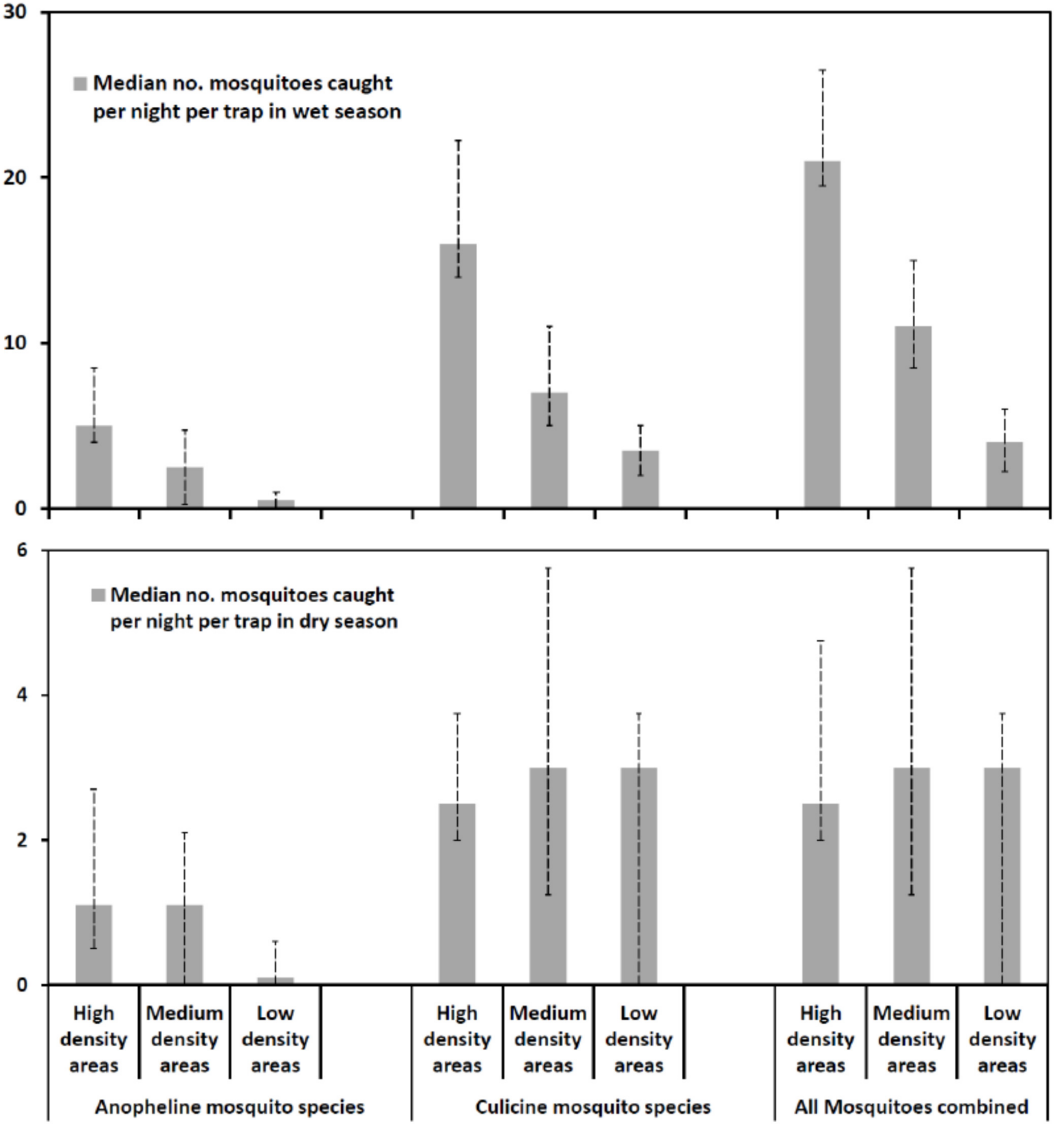
Comparison of mosquito catches in areas classified by communities as having high, medium or low mosquito densities in dry season and wet season. Median nightly mosquito catches in areas marked by community members as having high mosquito densities, medium densities or low densities in all villages during wet season (upper panel), and dry season (lower panel). Data segregated by taxa, but combined over 12 months. The error bars in this graph represent the inter-quartile ranges, i.e. 25^th^ percentile and 75^th^ percentile on either side of the median nightly catch. Data for the wet season included months of December, January, February, March, April and May, while the dry season data included June, July, August, September, October and November.

We also segregated the data by taxa and examined trends of anopheline mosquitoes (including the two main malaria vectors in the area, i.e. *An*. *arabiensis*, and *An*. *funestus*, but also all other minor *Anopheles* species such as *An*. *coustani*) relative to trends of culicine mosquitoes (including primarily *Culex* mosquito species and *Mansonia* species). Fig 8 shows the differences in median nightly trap catches of mosquitoes in different taxa in both wet season, and dry season. Entomological assessments revealed similar trends of mosquitoes in both dry and wet season, even though in the wet season, these differences were apparent only with anopheline mosquitoes but not with culicines. Mosquito numbers were significantly lower in the dry season than in the wet season (P < 0.001), and the differences in catches between locations was more apparent in dry season.

### Monthly variations in mosquito catches in areas considered by community members as having high, medium or low mosquito densities

**Fig 9:**
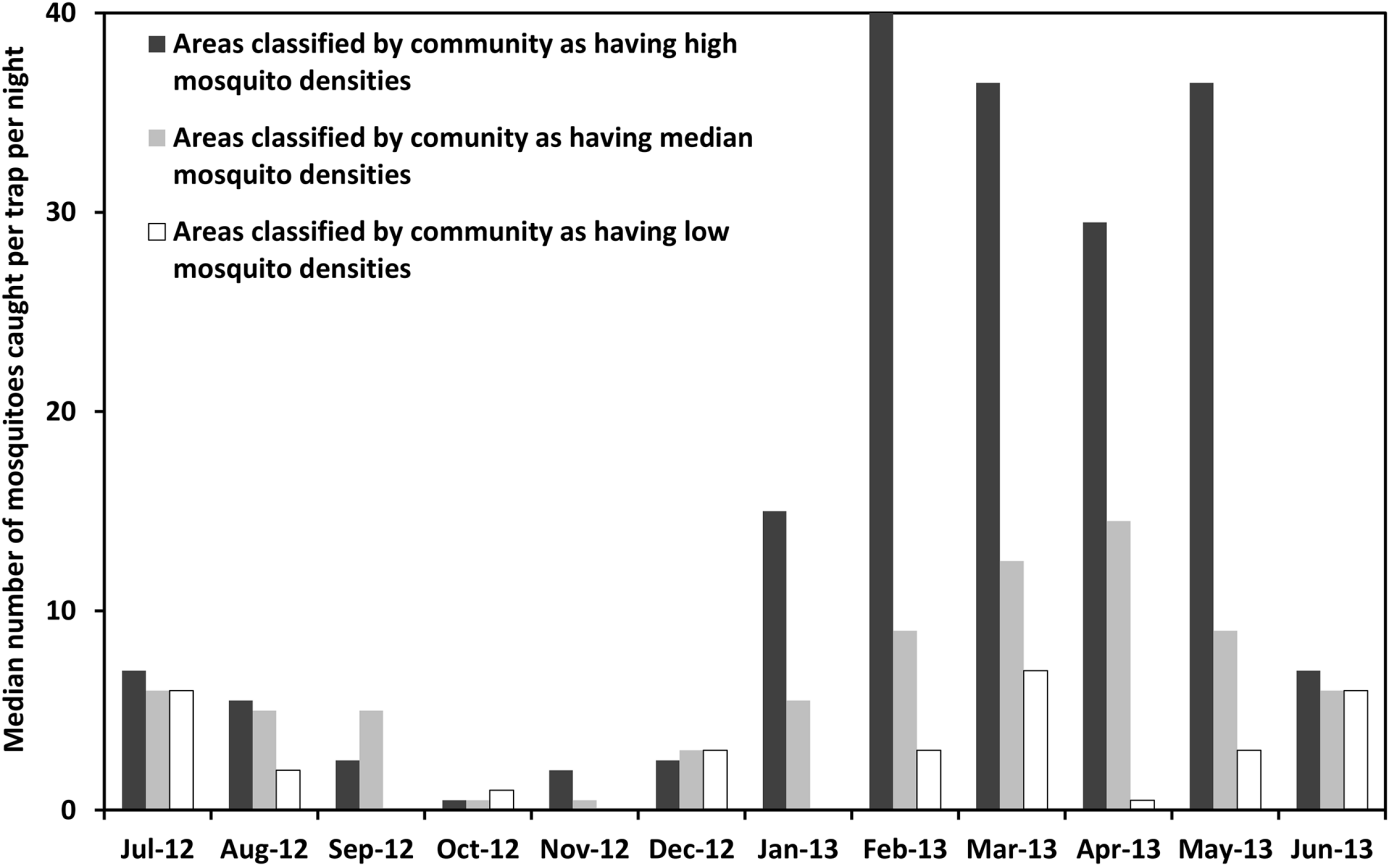
Monthly comparisons of mosquito catches in areas classified by communities as having high, medium or low mosquito densities. Month by month variations of nightly mosquito catches in areas marked by community members as having high mosquito densities, medium densities or low densities in the different months of collection, between July 2012 and June 2013. Data aggregated for all three study villages.

Community members correctly identified areas with most abundant mosquito densities and areas with least abundant densities for all the months starting July 2012 to June 2013. Analysis of the combined data from all villages (Fig 9) illustrated that mosquito catches were higher in wet season, peaking between February and May, than the rest of the period. Since we had conducted the entomological assays monthly, we could verify community opinions on for all the months. The highest mosquito catch was observed between February and May 2013, while the lowest densities were in october and November 2012in all categories as classified by community members (Fig 9). The overall results demonstrate that community members in these three rural Tanzanian villages could accurately identify areas where mosquitoes were most abundant, as well as areas where mosquitoes were least abundant, based solely on their knowledge and experiences. Fig 9 also illustrates that the ability of the community members to identify such locations, was consistent throughout the year, though it was less apparent in dry season than in wet season.

## Discussion

New approaches for identifying areas where disease-transmitting mosquito are most abundant and where exposure to mosquito-borne pathogens is highest could guide disease control responses and enable optimization of resource allocation, therefore maximizing potential benefits while minimizing costs. The aim of this study was to demonstrate the use of readily available knowledge and experiences of local community residents to predict density and distribution of disease-transmitting mosquitoes. Though our studies were conducted in three contiguous rural villages in south-eastern Tanzania, the findings could potentially be applicable in several other rural and remote communities where specific infectious diseases are endemic. However, we propose that additional studies would be necessary to validate this approach in multiple localities before its wide acceptance.

**Fig 10:**
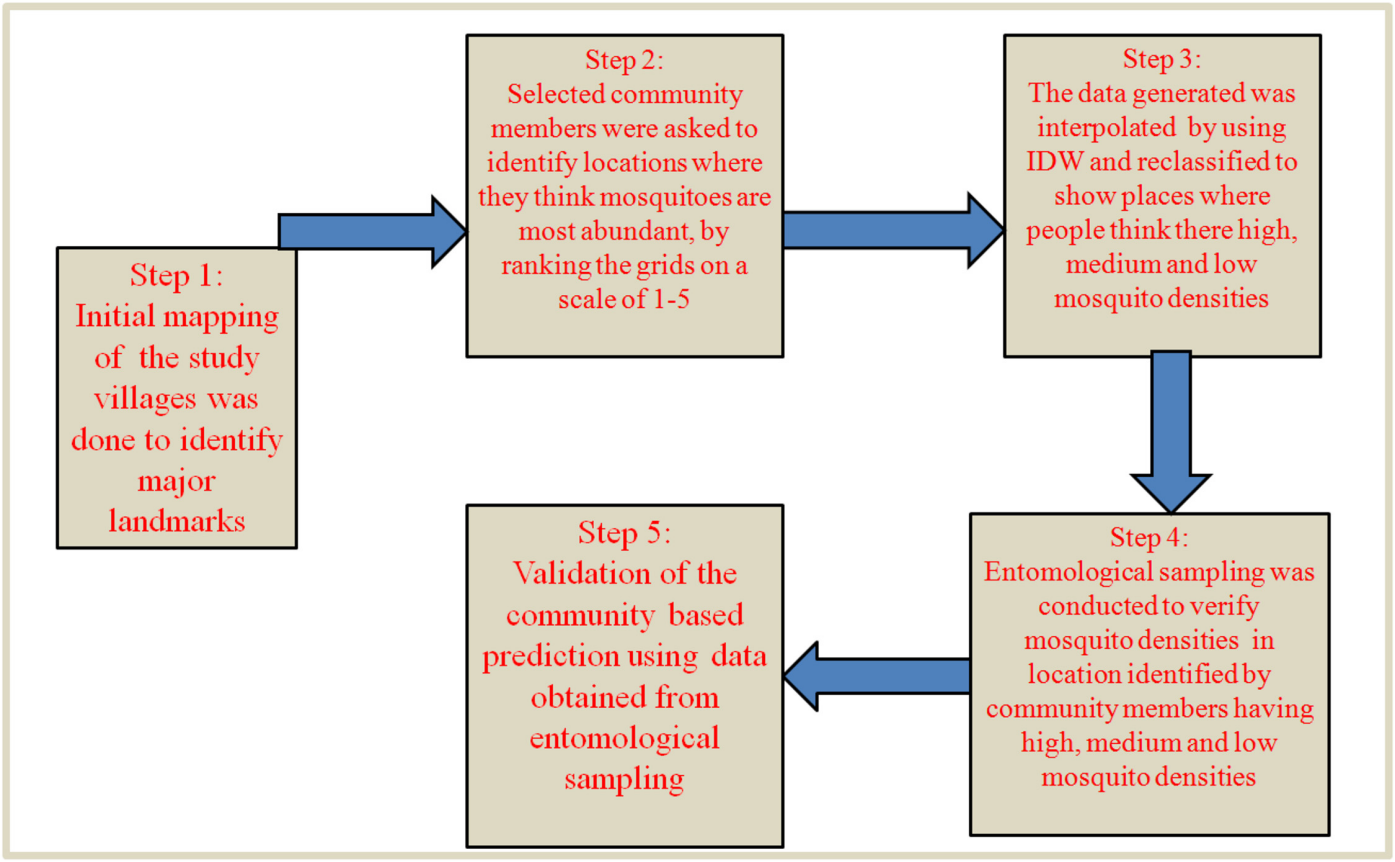
Main stages in the process of crowdsourcing vector surveillance. Illustration of the five main steps when crowdsourcing for community knowledge and experiences to predict or approximate densities and distribution of outdoor-biting mosquitoes.

Our hypothesis was derived from an earlier smaller study conducted in one village, also in rural Tanzania but in a single dry season. We worked with the basic assumption that people (including children and adults) who have lived in certain communities for reasonably long periods of time, would remember certain key features, possibly including features associated with increased mosquito bites, and that these people, if prompted, would be able to represent the features visually on maps of their own communities [1]. The only requirement therefore would be that the people are guided through a process of map reading and interpretation of essential map features. Secondarily, this study approach was also meant to increase our level of community engagement, and to ensure that community members in these study villages were more aware of the research work that we conducted in their communities. This approach is therefore fully community-based, is highly participatory and relies primarily on the knowledge and experiences of residents to rapidly identify areas where disease-transmitting mosquitoes are most abundant, so that public health officials can take necessary action. The method is simple, easy to implement, low-cost and directly involves community members to address the problems in the surrounding areas. As shown in Fig 10, the approach involves five simple steps, which can be completed within weeks, with only a small number of skilled personnel.

Results from the three study villages over the 12 month-period show clearly that when community members, including adults and school children were provided with gridded maps of their own villages, they were able to identify and rank locations where outdoor-biting mosquito densities were most abundant, simply by ranking the grids. Their ability to rank grids on a simple scale of 1–5 enabled us to further differentiate these locations and re-classify entire villages into categories as high density category, medium density category and low density category, at any time during the year, including both dry and wet seasons. The community knowledge and experiences clearly depicted not only the spatial but also temporal patterns in variations of vector densities. We showed that we can rely on community knowledge and experiences to identify areas where mosquitoes are most abundant or least abundant, even without entomological surveys. However, we also note that this crowd-sourcing method, though simple, may need to be further refined and validated to improve community-based planning of mosquito control operations at low-cost.

The technique we have described can be described as a form of crowdsourcing, which is a term commonly used in business, but refers simply to a set of procedures or processes for gathering ideas, new information or techniques from a community of people, regardless of whether the people are considered experts or not. Simply, it is the use of community-driven know-how to solve problems. Crowd sourcing approaches have already been widely applied in other sectors, mostly relying on online transmission of data [49], but are not yet popularly used in health research or intervention planning. In previous cases, such approaches have been successfully for mapping pollution levels in cities [50], improving efficiencies in surface transportation [51], for enhancing relief operations during disasters [17,52,53] and for marketing [19], among other applications.

In recent years, similar applications of such community-driven know-how have been increasing in health care practice and research. Ranard *et al*., recently provided a systematic review of how knowledge of the masses has been and could be harnessed to advance medicine [18]. Other examples include using mobile phones for community based health reporting, in what is now called, participatory epidemiology [54], crowd sourcing malaria parasite quantification [55] and interestingly, crowd sourcing medical expertise in near-real time [56]. While our approach relied on analogue rather than digital data collection methods, so that we could capture information from all community members in rural areas without smartphones and computing capabilities, we have demonstrated that similar crowd sourcing approaches could be considered and improved to support mapping of densities of disease-transmitting mosquitoes.

We propose use of this approach could be further tested and validated to assess its potential for spatial targeting of mosquito control interventions, which is an increasingly important concern particularly in malaria control, where residual transmission is thought to be increasingly mediated by mosquitoes that bite outdoors, but also because of the need to provide complementary interventions alongside the current primary interventions such as LLINs and IRS, mostly used indoors [31]. If proven, the approach would also be useful for planning implementation of other large-scale mosquito abatement operations such as larval source management [57] and even aerial mosquito spraying [58]. Such simplified methodologies for mapping outdoor vector densities will be particularly necessary for optimal placement of outdoor mosquito control devices, such as odour-baited mosquito traps or lure and kill stations, which have been suggested to be more effective if preferentially located in areas with highest mosquito densities [59]. In our study, the number of geographical grids identified by community members as having either high or medium vector densities throughout the study period was consistently a small fraction of the total area, suggesting that the principles of heterogeneous distribution of risk as described by Woolhouse et al [60] and Smith *et al*., [61], still hold, even when we rely primarily on local community knowledge and experiences. Results of such participatory mapping approaches could therefore be used to improve or fine-tune existing approaches for targeting pockets of high vector densities. Where high vector density areas overlap with pockets of high pathogen transmission, it may then be reasonable to target these for improved disease control [30]. Even though the crowd-sourcing approach that we used here was not designed in a way that would allow distinguishing different times of the year or even different mosquito species, the results suggest that both these may be possible, but would require further refinements of the techniques.

While we observed the differences most clearly when total mosquito catches were considered in the different analyses, the differences were also observable when the mosquitoes were classified by taxa. Even though we did not ask the community members to distinguish locations with various densities of different taxa, there was an apparent match between the opinions of the residents and the entomological surveys across taxa. Particularly interesting was that the densities of host seeking anophelenes, which in this case predominantly consisted of malaria mosquitoes, *An*. *arabiensis* and *An*. *funestus* group, also followed the same pattern and matched community representations in all the three villages. This indicates that either the community members probably relied mostly on their knowledge of human-biting mosquitoes, which in this area are mostly represented by the *Anopheles* mosquitoes. Alternatively, the fact that anopheline trends also matched community opinions may be due to a situation that mosquito species in the area have overlapping ecological niches and habitats, which is indeed the more plausible explanation.

Our direct observations also revealed that the community members had predicted areas with feature such as cultivated rice fields, road junctions, market places and high density of households, even though these features were not consistently obvious in all selections in all villages. We can infer the likelihood that the people made their predictions and rankings based on their associations of high mosquito densities with features such as surface water bodies, (streams, rivers and cultivated rice fields) and high human population densities (household locations and markets, churches, mosques and road junctions). These places where humans congregate in evenings were more likely to be predicted as having high mosquito densities because people do indeed experience bites when they congregate in these locations, and mosquito species, particularly malaria vectors preferentially seek and bite humans over other hosts. Nonetheless, the results suggest that the improved versions of this crowd-sourcing methodology could in future be appropriate for both generalized and species-targeted surveillance and response operations against mosquitoes.

This technique is very simple and enables rapid for delineation of high-risk or low-risk areas, with clear temporal depiction or patterns in mosquito distribution. Nonetheless, the actual procedures that we have presented here can and should be improved further. Examples of such improvements may include: 1) improving methods of collecting community opinions through use of mobile phones, 2) improving the methods of interpolation and classification of community data, or 3) by combining the final product with other data sets like digital aerial maps to produce even more accurate outcomes. Though not tested here, additional advantages of this approach would include improved community engagement and greater participation of community members in planning for vector control. We also propose that future studies would be necessary to validate these results by investigating potential of such a technique in far distant villages in multiple districts, unlike in this current study where we worked in contiguous villages. We recognize that article provides only the very first demonstration of the potential of community members’ experiences and knowledge, and that the findings of our study will need to be validated by other field studies elsewhere to eventually demonstrate the true value of this approach.

### Conclusion

The challenge of identifying areas where mosquito biting risk is highest or lowest within communities can be solved by relying on the knowledge and experiences of young and adult residents in those communities. At this stage, this study provides clear field evidence that we can rely on community knowledge and experiences to identify areas where mosquitoes are most abundant or least abundant, even without entomological surveys. We expect that the approach could be refined and validated in other study sites to improve community-based planning of mosquito control operations at low-cost. Visual maps produced on the basis of community knowledge and experiences were directly verified using comparative entomological surveys over 12 months in three different villages as accurate. Such methods would be low-cost, quick and easy to use, and could potentially guide large scale implementation of mosquito control operations. The methodology used is very simple and rapid for delineation of high-risk or low-risk areas, with temporal patterns of mosquito distribution. However, we recommend that the methods can and should be enhanced by: 1) improving methods of collecting community opinions through use of mobile phones, 2) improving the methods of interpolation and classification of community data, or 3) by combining the final product with other data sets like digital aerial maps to produce even more accurate outcomes.

## Supporting Information

S1 File Supplementary maps. A folder containing supplementary maps depicting interpolated maps of locations identified by community members as having high, medium or low mosquito densities in Kivukoni, Mavimba and Minepa villages.
(RAR)
